# Supplementary material for: A Seven-Year Microbiological and Molecular Study of Bacteremias Due to Carbapenemase-Producing Klebsiella Pneumoniae: An Interrupted Time-Series Analysis of Changes in the Carbapenemase Gene’s Distribution after Introduction of Ceftazidime/Avibactam
Source: Antibiotics (Basel). 2022 Oct 14;11(10):1414. doi: 10.3390/antibiotics11101414 (PMC9598502; doi:10.3390/antibiotics11101414)
Supplement: Supplementary file 1 [file antibiotics-11-01414-s001.zip › Supplementary Table S1.pdf]

Supplementary Table S1. MIC distribution and susceptibility of 499 carbapenemase-producing *K. pneumoniae* isolates to different antimicrobials according to EUCAST guidelines

**Antibiotics**	**MIC (mg/L)**																	**EUCAST**	
	**0.032**	**0.064**	**0.094**	**0.125**	**0.25**	**0.38**	**0.5**	**0.75**	**1**	**1.5**	**2**	**3**	**4**	**6**	**8**	**12**	**32**	**S (%)**	**R (%)**
Colistin		3	1	18	36	10	91	38	64	41	46	*14*	*21*	*14*	*25*	*66*	*11*	348 (69.7)	151 (30.3)
Tigecycline		1		4	21	24	54	*63*	*86*	*60*	*83*	*26*	*32*	*12*	*23*	*13*	*6*	104 (20.8)	395 (79.2)
Ceftazidime/avibactam[a]	9	4	2	7	7	19	33	18	8	6	1	1	4	1			*15*	118 (88.7)	15 (11.2)
	**1**	**2**	**3**	**4**	**6**	**8**	**12**	**16**	**24**	**32**	**48**	**64**	**96**	**128**	**256**	**512**	**1024**	**S (%)**	**R (%)**
Fosfomycin[b]		5	4	6	6	18	31	30	18	27	*9*	*12*	*6*	*7*	*4*	*2*	*32*	145 (66.8)	72 (33.2)

Resistant isolates according to EUCAST appear in italics

[a]Only for 133 *K. pneumoniae* isolates of 2018-19 carrying *bla*KPC

[b]Only for 217 *K. pneumoniae* isolates
