# Supplementary material for: A Seven-Year Microbiological and Molecular Study of Bacteremias Due to Carbapenemase-Producing Klebsiella Pneumoniae: An Interrupted Time-Series Analysis of Changes in the Carbapenemase Gene’s Distribution after Introduction of Ceftazidime/Avibactam
Source: Antibiotics (Basel). 2022 Oct 14;11(10):1414. doi: 10.3390/antibiotics11101414 (PMC9598502; doi:10.3390/antibiotics11101414)
Supplement: Supplementary file 1 [file antibiotics-11-01414-s001.zip › Supplementary Table S2.pdf]

Supplementary Table S2. Distribution of *K. pneumoniae* BSIs according to hospital department and presence of carbapenemase genes.

Species	All (n=632)	Emergency room (n=54; 8.5%)	Medical wards (n=163; 25.8%)	Haematology wards (n=74; 11.7%)	Surgical wards (n=108; 17.1%)	ICU (n=210; 33.2%)	Pediatric wards[a] (n=23; 3.6%)
Carbapenem-susceptible	133 (21.0%)	25 (46.3%)	50 (30.7%)	14 (18.9%)	26 (24.1%)	9 (4.4%)	9 (39.1%)
Carbapenem-resistant	499 (79.0%)	29 (53.7%)	113 (69.3%)	60 (81.1%)	82 (75.9%)	201 (95.6%)	14 (60.9%)
bla_{KPC}	384 (60.8%)	24 (44.4%)	81 (49.7%)	58 (78.4%)	59 (54.6%)	148 (70.5%)	14 (60.9%)
bla_{NDM}	61 (9.7%)	5 (9.3%)	24 (14.7%)	2 (2.7%)	12 (11.1%)	18 (8.6%)	0 (0.0%)
bla_{VIM}	39 (6.2%)	0 (0.0%)	4 (2.5%)	0 (0.0%)	10 (9.3%)	25 (11.9%)	0 (0.0%)
bla_{KPC} and bla_{VIM}	12 (1.9%)	0 (0.0%)	2 (1.2%)	0 (0.0%)	1 (0.9%)	9 (4.3%)	0 (0.0%)
bla_{NDM} and bla_{VIM}	3 (0.5%)	0 (0.0%)	2 (1.2%)	0 (0.0%)	0 (0.0%)	1 (0.5%)	0 (0.0%)

ICU: Intensive Care Unit

[a]including neonatal and paediatric ICU
