# Supplementary figures and images for: A Seven-Year Microbiological and Molecular Study of Bacteremias Due to Carbapenemase-Producing Klebsiella Pneumoniae: An Interrupted Time-Series Analysis of Changes in the Carbapenemase Gene’s Distribution after Introduction of Ceftazidime/Avibactam
Source: Antibiotics (Basel). 2022 Oct 14;11(10):1414. doi: 10.3390/antibiotics11101414 (PMC9598502; doi:10.3390/antibiotics11101414)

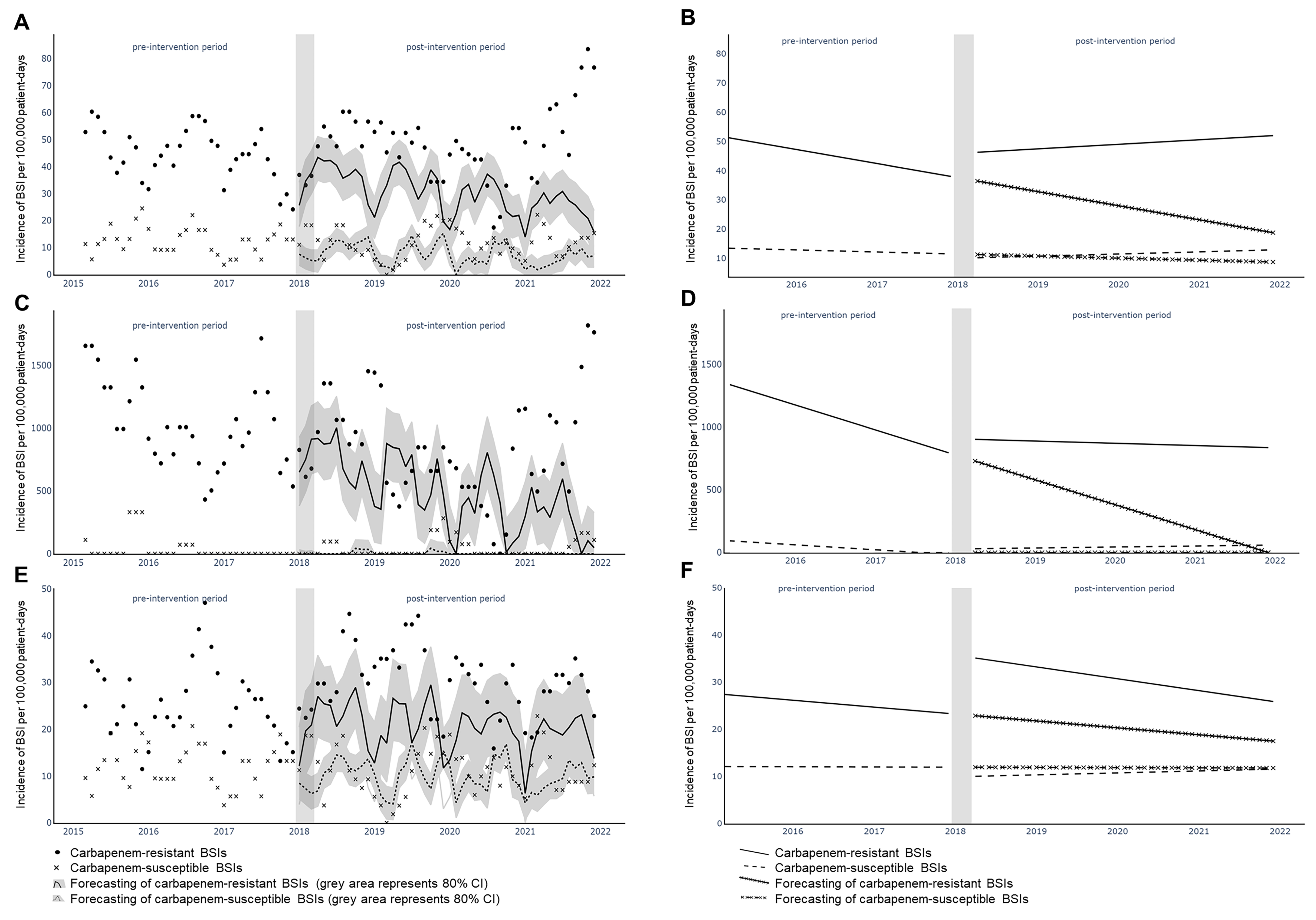

Supplement: Supplementary file 1 [file antibiotics-11-01414-s001.zip › Supplementary Figure S1.tif]

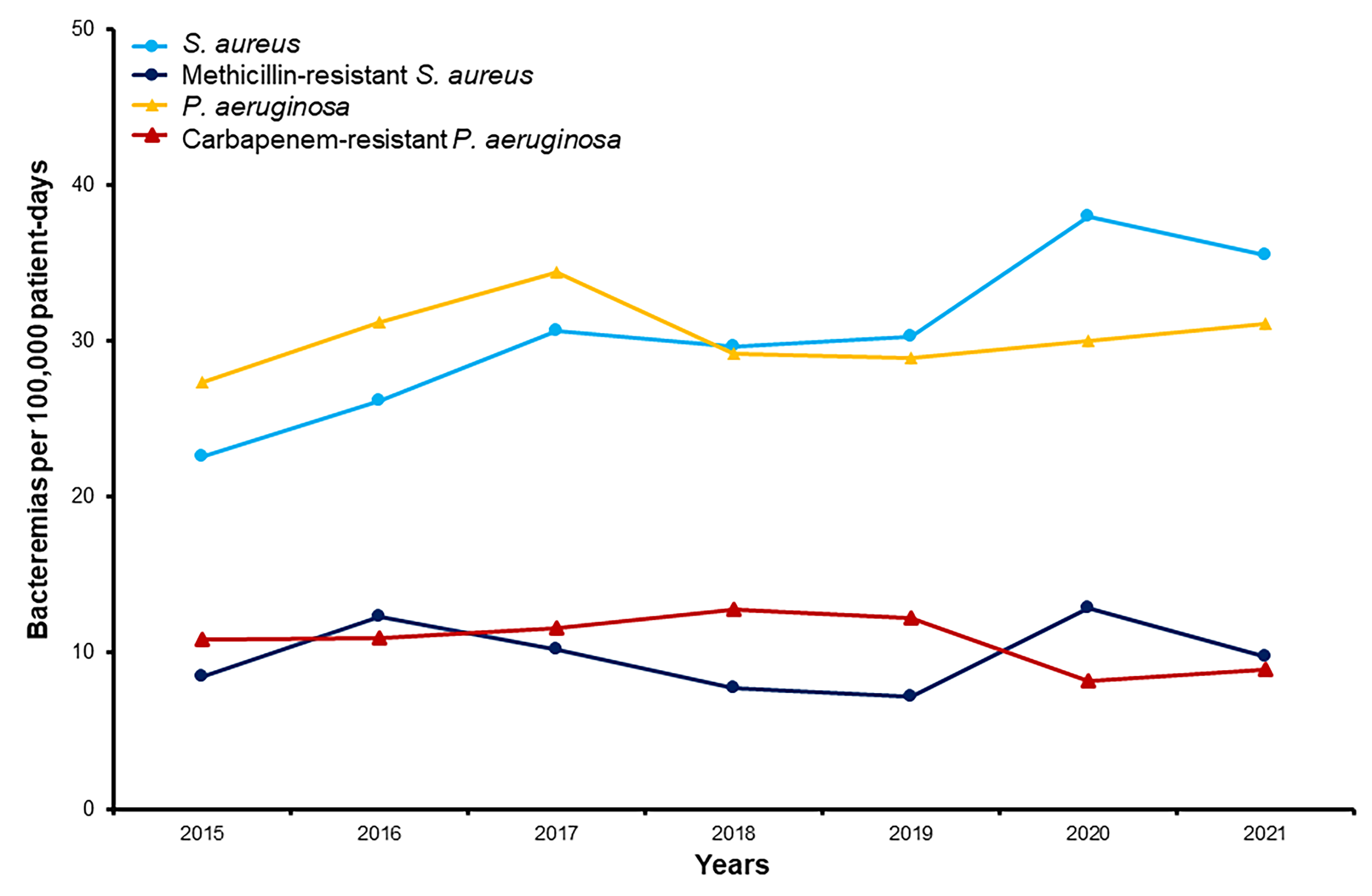

Supplement: Supplementary file 1 [file antibiotics-11-01414-s001.zip › Supplementary Figure S2.tif]
